# Cancer exosomes trigger mesenchymal stem cell differentiation into pro-angiogenic and pro-invasive myofibroblasts

**DOI:** 10.18632/oncotarget.2711

**Published:** 2014-11-28

**Authors:** Ridwana Chowdhury, Jason P. Webber, Mark Gurney, Malcolm D. Mason, Zsuzsanna Tabi, Aled Clayton

**Affiliations:** ^1^ Institute of Cancer and Genetics, School of Medicine, Cardiff University, Velindre Cancer Centre, Whitchurch, Cardiff, United Kingdom, CF14 2TL; ^2^ Cardiff Institute for Tissue Engineering and Repair, Cardiff university

**Keywords:** exosomes, cancer stroma, mesenchymal stem cells, prostate cancer

## Abstract

Stromal fibroblasts become altered in response to solid cancers, to exhibit myofibroblastic characteristics, with disease promoting influence. Infiltrating mesenchymal stem cells (MSC) may contribute towards these changes, but the factors secreted by cancer cells that impact MSC differentiation are poorly understood.

We investigated the role of nano-metre sized vesicles (exosomes), secreted by prostate cancer cells, on the differentiation of bone-marrow MSC (BM-MSC), and the subsequent functional consequences of such changes. Purified exosomes impaired classical adipogenic differentiation, skewing differentiation towards alpha-smooth muscle actin (αSMA) positive myofibroblastic cells. A single exosomes treatment generated myofibroblasts secreting high levels of VEGF-A, HGF and matrix regulating factors (MMP-1, −3 and −13). Differentiated MSC had pro-angiogenic functions and enhanced tumour proliferation and invasivity assessed in a 3D co-culture model. Differentiation was dependent on exosomal-TGFβ, but soluble TGFβ at matched dose could not generate the same phenotype. Exosomes present in the cancer cell secretome were the principal factors driving this phenotype.

Prostate cancer exosomes dominantly dictate a programme of MSC differentiation generating myofibroblasts with functional properties consistent with disease promotion.

## INTRODUCTION

Tumourigenesis is a multicellular process dependant on intercellular communication and other extracellular signals within the microenvironment [1]. Genetically transformed cells can manipulate normal cells within the milieu, and are reciprocally responsive to such changes in a bidirectional manner. A particularly important relationship exists between tumour and stromal fibroblast-like cells, where the proportion of stromal cells is often elevated together with changes in stromal cell activation states [1–2]. Solid tumours including those of prostate, breast, colon and others reveal an accumulation of myofibroblastic stromal cells [3] that exert a powerful influence on disease initiation, progression, treatment response and ultimately, prognosis [4–8]. A better understanding of the cross-communication between tumour and stromal cells is likely to lead to progress in the clinical management of diverse solid tumours.

Cancer associated stroma drive remodelling of tissue architecture by altering matrix, and by producing assorted growth factors that directly promote tumour growth [9–10], and promote processes such as angiogenesis [10–11] and metastasis [12]. However, the cellular origin of stromal myofibroblasts remains unclear [13], with an assortment of possibilities that include tissue resident fibroblasts or vascular pericytes, or through epithelial or endothelial transition to mesenchymal phenotypes [14]. There may also be contributions from infiltrating cells like mesenchymal stem cells of adipose or bone marrow origins [15–16].

Mesenchymal stem cells are defined by the international society for cellular transplantation (ISCT) as adherent cells positive for CD73, CD90, CD105, CD146 and negative for haematopoietic markers like CD14 or CD45 [17]. MSC are multipotent and are capable of generating the various cell types of connective tissue including; myocytes, neurones, osteoblasts, chondrocytes, adipocytes and fibroblasts. Their physiological roles include migration into, and repair of inflammatory/injured tissues [18], and some studies aim to manipulate this property as a therapeutic intervention [19]. The intra-tumoural migratory potential of MSC however, has been difficult to assess in humans [20], but migration into tumour xenografts, following intra venous administration has been documented for prostate [21], colon [22], breast [23–24] and other murine cancers models. The presence of MSC in human cancer tissue has certainly been documented, estimating a proportion of < 1.1% of the total cells in prostate for example [21]. In general, MSC which naturally arise at tumour sites or those co-administered with tumours in model systems exhibit similarities to cancer associated stroma in their tumour-promoting effects [15–16, 24–25].

Under the influence of secreted factors of tumour cell origin, MSC can undergo differentiation into myofibroblast-like cells [24]; akin to those of cancer-associated stromal cells, and it is this form of differentiation that is related to their tumour-promoting effect(s) [24–25]. However the factors within the cancer cell secretome responsible for driving this particular differentiation programme remain incompletely understood. Some recent studies point to a potential role for small extracellular vesicles, called exosomes, which are secreted in relative abundance by cancer cells. Such vesicles are pre-manufactured within late endosomal compartments called multivesicular endosomes. These are transported to, and fuse with, the plasma membrane resulting in vesicle release into the extracellular space [26]. Regulators of exosome biogenesis and trafficking within cells are poorly understood but some Rab-family members have been implicated in trafficking. Of these, Rab27a and Rab27b are the most extensively studied to date, as knockdown of these proteins offer a means of selectively inhibiting exosome secretion by various cell types [27]. Our recent studies showed cancer exosomes can act to modulate stromal cell fate, driving fibroblast to myofibroblast differentiation, in a manner requiring exosomally-tethered TGFβ1 [28], and that such myofibroblasts had potent angiogenic and tumour promoting properties *in vivo*; effects that were lost when using Rab27a knockdown cancer cells [29]. Exosomes from other cancer types may also impart similar effects on driving a myofibroblast-like differentiation process, and MSC of adipose or cord-blood origin have been reported to be responsive to exosomally delivered TGFβ1 [30–32].

In the current report, therefore, we examined the possible influence of prostate cancer derived exosomes on bone marrow derived MSC, and in turn the reciprocal influence of modified MSC on endothelial cells and tumour cells. We demonstrate exosomes play a role in controlling BM-MSC differentiation to myofibroblasts, through a TGFβ1 dependent mechanism, producing cells that exhibit heightened VEGF, HGF and metalloproteinases. Exosome-differentiated MSC in turn enhanced tumour and endothelial cell proliferation and migration, supported endothelial vessel formation and tumour invasion *in vitro*. Prostate cancer exosomes can therefore dictate the fate of MSC in a dominant manner, generating myofibroblastic cells with tumour promoting characteristics.

## RESULTS

### Characterisation of BM-MSC

**Figure 1:**
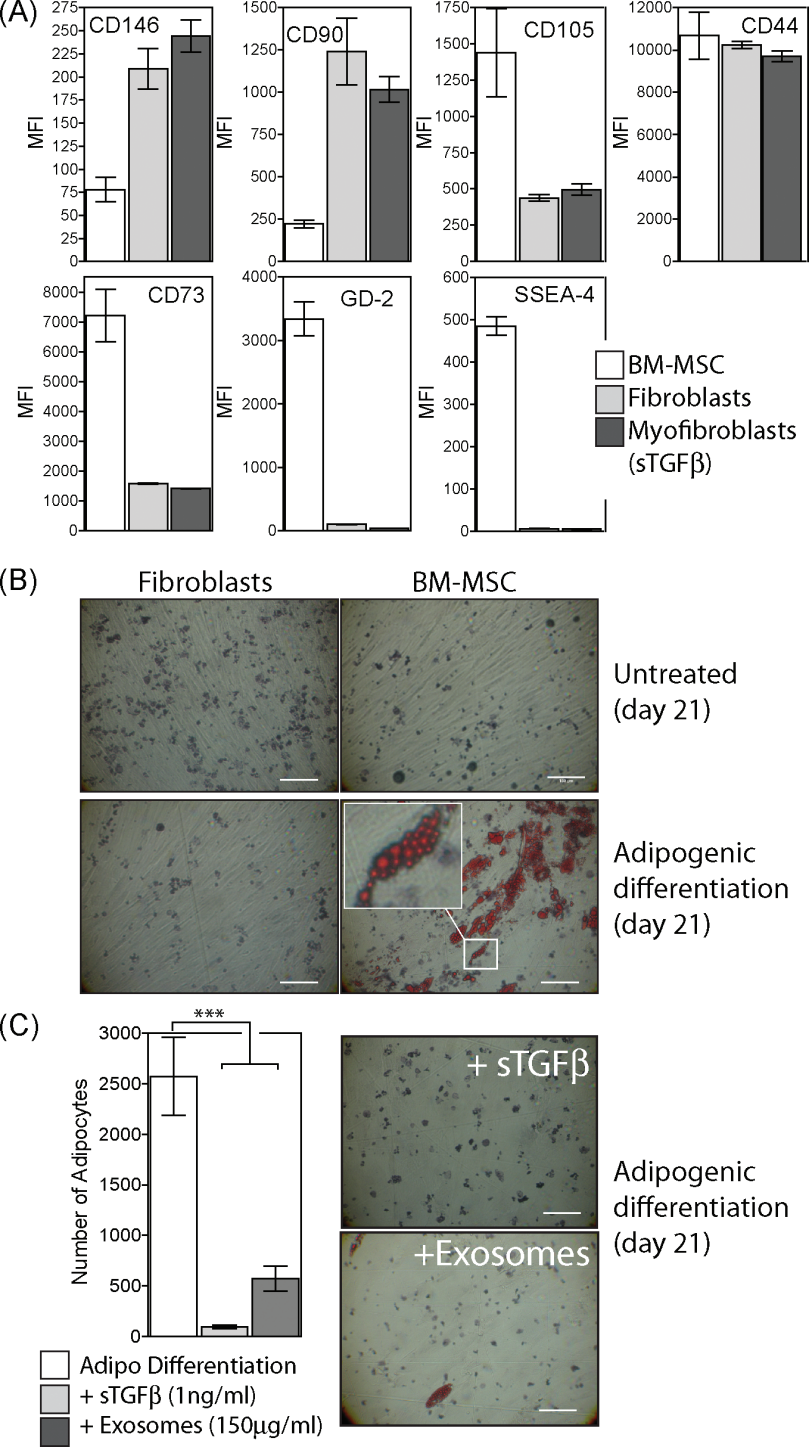
Characterisation of BM-MSC phenotype and differentiation Commercially obtained BM-MSC (at passage 2) were analysed by flow cytometry for a range of markers as depicted. These cells were compared to fibroblasts or to myofibroblasts generated following 3d treatment of fibroblasts with sTGFβ (1ng/ml). Bars show median fluorescence intensities (MFI) of duplicate measurements **(A)**. Culture of BM-MSC or fibroblasts under conditions favouring adipogenic differentiation for 21 days was followed by staining with Oil Red O (Scale, 100μm) **(B)**. Selection from image showing clusters of Oil Red O-stained fat droplets in adipocytes (B, inset). During adipogenic differentiation, sTGFβ (1ng/ml) or prostate cancer exosomes (150 μg/ml) were repeatedly added and the formation of Oil Red O positive adipocytes examined at day 21. Bars show the mean (± SD) number of adipocytes per field of view, from a total of 10 microscopic fields examined in duplicate wells per treatment, and are representative of two independent experiments **(C)**.

We analysed the surface-phenotype of commercially obtained BM-MSC according to the classical ISCT criteria, comparing them to fibroblasts and myofibroblasts. Although the BM-MSC well satisfied the expected characteristics of positive expression for the markers CD146, CD90, CD104, CD44 and CD73 and negative for CD45 (not shown), the other types of stromal cells analysed also displayed these characteristics (Fig. 1A). This panel of markers alone cannot therefore distinguish MSC from fibroblasts, or from myofibroblasts generated by 72 h treatment of fibroblasts with soluble TGFβ1 (at 1ng/ml). However, positive staining for the embryonic stem cell marker SSEA-4, and the Ganglioside GD2 previously reported as MSC markers [33], was absent from other stromal cell types, demonstrating BM-MSC are phenotypically distinct from other stromal types.

We next examined the capacity of these cells to undergo a classical form of MSC-differentiation, namely into adipocytes. Following culture in appropriate conditions for a period of 21 days, BM-MSC developed multiple lipid droplets within the cytosol, stained intensely with Oil red O (Fig. 1B). Fibroblasts treated identically showed no signs of adipogenic differentiation.

Exosomes were purified from prostate cancer DU145 cells and were previously shown to contain around 7pg of TGFβ1 per μg of exosomes [28, 34]. These were repeatedly added (at 150 μg/ml) to some wells together with adipogenic differentiation factors every 3 days throughout the 21 day experiment. This dose of exosomes is approximately equivalent to 1ng/ml of TGFβ1. Alternatively, soluble TGFβ1 (at 1ng/ml) was added instead of exosomes, and the effects on adipogenesis were compared. Either treatment resulted in significant (*P* < 0.001) inhibition of differentiation into adipocytes (Fig. 1C).

The data demonstrate that the BM-MSC used in the study were consistent with expectations of MSC phenotype and differentiation capacity, but crucially the differentiation fate of these cells can be modulated by cancer-derived exosomes which are capable of overriding the adipogenic differentiation programme.

### Exosome treated BM-MSC become myofibroblast-like

**Figure 2:**
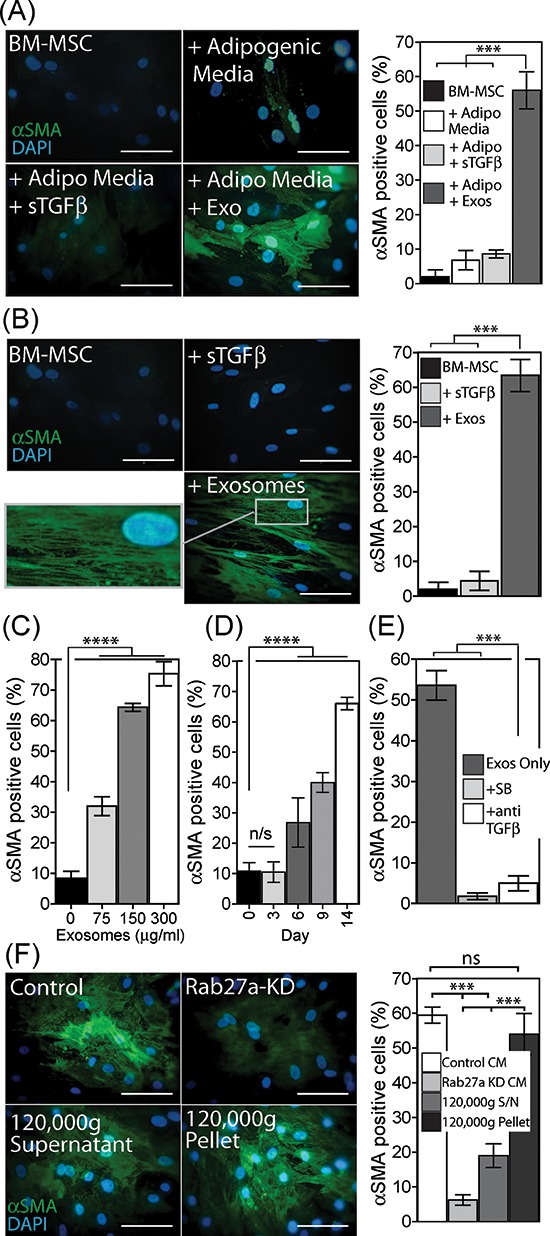
Exosomes drive differentiation of BM-MSC to a myofibroblast-like phenotype Under adipogenic conditions, exosomes (150 μg/ml) or sTGFβ (1ng/ml) were added to BM-MSC, as depicted and at 21 days the cells were stained for αSMA (green) and DAPI (blue). Quantitation of the proportion of αSMA positive cells, from a total of 6 microscopic fields examined in duplicate wells per treatment, is shown. Representative of two independent experiments **(A)**. A single treatment with exosomes or sTGFβ, at the above dose, in the absence of adipogenic differentiation factors was performed, and at day 14 cells were stained and quantified for αSMA positive cells as above. Representative of 5 such experiments **(B)**. BM-MSC were stimulated for 14 d with increasing doses of exosomes (0–300 μg/ml), and the proportion of αSMA positive cells were counted as above **(C)**. Similarly at a fixed dose of exosomes (150 μg/ml), the proportion of αSMA actin positive cells were determined at time points up to 14 days **(D)**. BM-MSC were treated with exosomes (150 μg/ml) in the absence or presence of the Alk-5 inhibitor (SB431542) or neutralising antibody against TGFβ, and at day 14, αSMA expression quantified as above **(E)**. Culture medium normalised for cell number, was taken from control or Rab27a^KD^ Du145 cancer cells, or from control Du145 cells following ultracentrifugation to pellet exosomes (120,000g supernatant), or the exosome-containing pellet from this spin was resuspended in the original volume and used (120,00g pellet). These CM were added to BM-MSC and at day 14 αSMA expression was quantified as above, representative of two experiments. **(F)** (Scale, 100 μm, Bars, Mean ± SD).

We examined whether or not BM-MSC respond to exosomes by differentiating into a phenotype similar to that of cancer associated stromal cells as we described recently for the response of fibroblasts to exosomes [29]. Under adipogenic conditions, exosomes or sTGFβ were added during the 21-day differentiation period and the impact on expression of the myofibroblastic marker alpha-smooth muscle actin (αSMA) was examined. The proportion of αSMA positive cells remained low (<5%) under adipogenic differentiation conditions, and this was not altered following sTGFβ treatment. In contrast, more than 50% of the cells exhibited strong αSMA expression following treatment with exosomes at a matched TGFβ-dose (Fig. 2A).

A simpler experiment was next performed in the absence of adipogenic differentiation conditions, giving a single stimulation with exosomes or sTGFβ at day 0, and evaluating the outcome earlier at day 14. Here again exosomes but not sTGFβ drove a significant elevation in αSMA positive cells (Fig. 2B), with the majority becoming positive for αSMA. Importantly αSMA protein was not simply elevated in these experiments but was present as organised stress-fibres (Fig. 2B); the onset of which is a key characteristic of myofibroblasts [35]. A single stimulus with exosomes was therefore sufficient to trigger myofibroblastic differentiation independently of any other differentiation factor. The response to exosome treatment was dose dependent, with an approximately 3 fold elevation, to ~30% of the population becoming αSMA positive at 75 μg/ml. This increased to around 75% with very high exosome doses of 300 μg/ml (Fig. 2C, and Fig. S1). The kinetics of αSMA onset however, was slower than we expected, certainly slower than that for fibroblasts in which αSMA peaks at around 72 h post exosome-stimulation [28]. There was no change in αSMA status by 72 h for BM-MSC, with changes only becoming apparent 6 days post exosome treatment but continuing steadily thereafter approaching 70% positivity by day 14 (Fig. 2D, Fig. S2).

Because the response of fibroblasts to exosomes is principally dependent on exosomal-TGFβ [28], we investigated the impact of interfering with TGFβ signalling using either an inhibitor of the Alk5 TGFβ-receptor-I (SB431542) or using a neutralising antibody against TGFβ that will bind to and inhibit exosomally-delivered TGFβ1 as we described [28–29]. Exosomes added with either inhibitor failed to trigger differentiation into αSMA-positive cells (Fig. 2E). The mechanism of action therefore requires exosomal-TGFβ driving signalling through TGFβ-receptor-I, yet this myofibroblastic phenotype cannot be reproduced using a matched dose of sTGFβ (Fig. 2A, 2B).

By adding purified exosomes to BM-MSC, however, it is difficult to evaluate if this effect is an artificial artefact due to the nature of the doses we have studied. We therefore investigated the role of exosomes naturally present within the cancer cell secretome by adding cancer cell conditioned media, normalised for cell number, to BM-MSC. For these experiments we used cancer cells that had been rendered deficient in exosome secretion by knockdown of Rab27a as described [27, 29]. Alternatively, we simply depleted exosomes from the conditioned media using ultracentrifugation. In the absence of such manipulations cancer cell conditioned media was capable of driving the differentiation of BM-MSC into αSMA-positive myofibroblasts (Fig. 2F). This was strongly inhibited when using cell medium from exosome deficient Rab27a^KD^ cells or when depleting the cell medium of exosomes by ultracentrifugation. With respect to the latter, the exosome-containing pellet resuspended in the original volume of medium was sufficient to fully restore differentiation. We conclude that exosomes and not other soluble factors within the cancer cell secretome are chiefly responsible for controlling the differentiation of BM-MSC into myofibroblasts.

### Exosome treated BM-MSC elevate pro-angiogenic factors and metalloproteinase's

**Figure 3:**
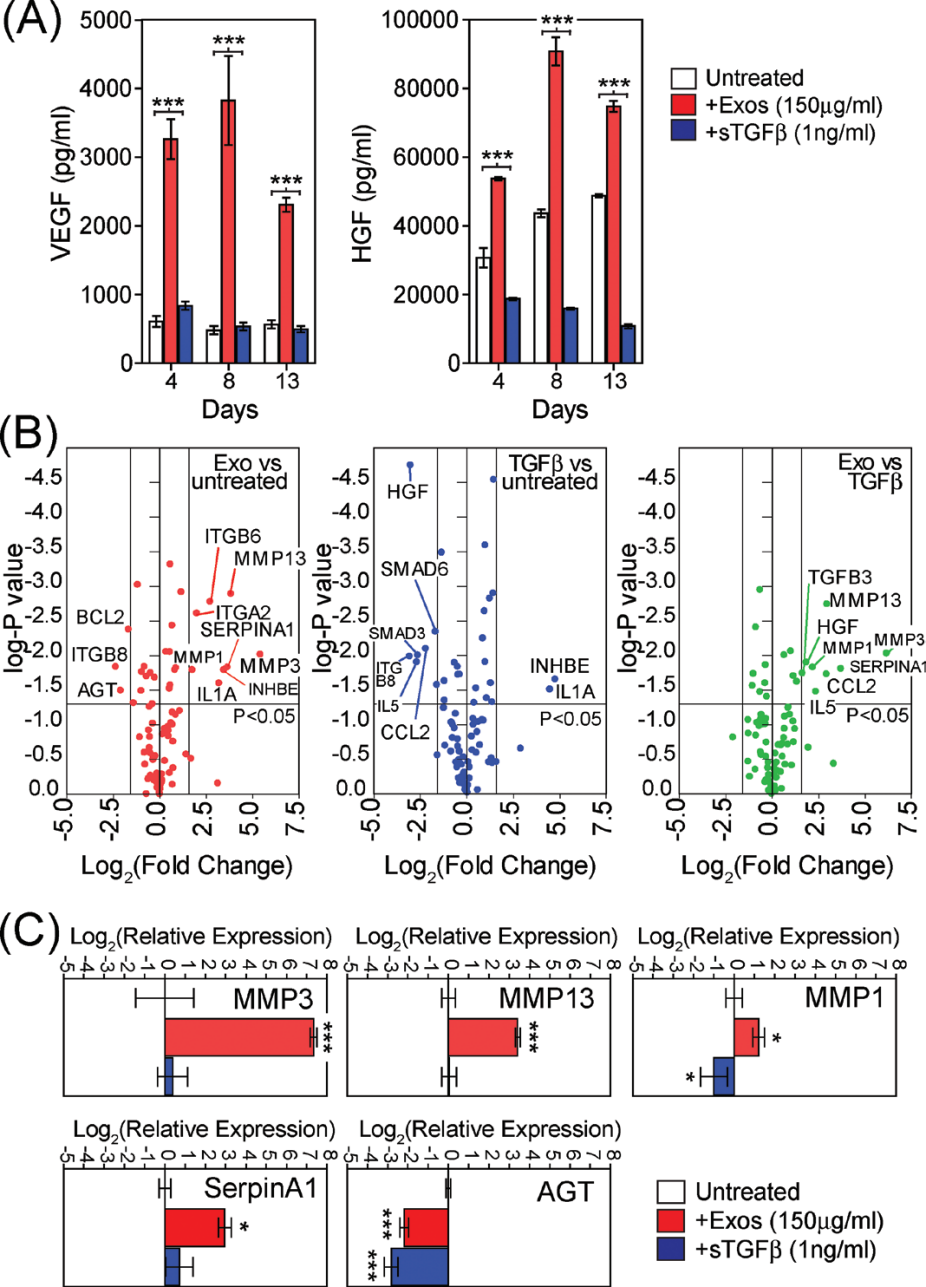
Phenotypic changes in exosome-differentiated BM-MSCs Conditioned media taken from BM-MSC treated with exosomes (150 μg/ml) or sTGFβ1 (1ng/ml) at specified time points, were analysed by ELISA for levels of VEGF (left) or HGF(right) **(A)**. Volcano plot, depicting results from RT^2^-Profiler™ fibrosis array comparing day4 untreated BM-MSC with exosome-treated MSC (B, left) or with sTGFβ treated exosomes (B, middle) or exosome-treatment vs sTGFβ treatment (B, right). Applied thresholds were a fold change of ± 3 and a *p*-value of ≤0.05 (t-test based on biological triplicates per treatment) **(B)**. TaqMan-PCR verification of selected transcripts identified by the array, revealing reproducible and significant changes in relative mRNA with GAPDH as an internal standard, at day 4. Columns represent Log_2_(relative expression) ± SD, compared to untreated BM-MSC (based on biological triplicates) **(C)**.

Because cancer associated stroma are implicated in supporting angiogenesis [29], we examined the possible effects of exosome-driven differentiation on angiogenic factors VEGF-A and HGF. A single stimulus with either exosomes or sTGFβ1 was given and an ELISA was used to measure the contents of BM-MSC conditioned media at several time points thereafter. There was a clear cut stimulation of VEGF secretion following exosome-treatment, which was more than 3-fold baseline levels after only 4 days, peaking at 8 days but still remaining elevated for the 2 week experiment (Fig. 3A, left). The levels here were far in excess of the VEGF-A added to the system in association with Du145-exosomes which we previously measured at 3pg VEGF-A per 1 μg of exosomes, giving 450pg/ml of VEGF-A added to the system [36]. Stimulating with sTGFβ had no impact on VEGF levels. There was a spontaneous rise in HGF levels from untreated BM-MSC, but this was still heightened following exosome treatment. Of note, sTGFβ treatment inhibited the spontaneous secretion of HGF (Fig. 3A, right), a phenomenon we also reported for fibroblasts [29]. Exosome stimulation therefore supports the production of pro-angiogenic proteins, and these alterations occur before the full onset of the αSMA-positive phenotype.

Due to these results, we also explored more broadly the consequence of exosome-stimulation on the BM-MSC phenotype arising. Using a focussed PCR-array method, with coverage of 84 transcripts of known involvement in tissue scarring/fibrosis-like reactions (Supp Table S1), we examined differential mRNA expression among the BM-MSC treated with exosomes or sTGFβ. Treatment with sTGFβ was not inert, as we saw elevated mRNA for IL-1A and INHBE, whilst there was a decrease in SMAD3, SMAD6, CCL2, IL5, ITGB8 and HGF compared to untreated BM-MSC (Fig. 3B, blue circles). Treatment with exosomes also elevated INHBE and IL1A, whilst decreasing ITGB8, but otherwise the alterations were dissimilar to those mediated by sTGFβ. Exosomes strongly elevated MMP-3, MMP-13, and SerpinA1 and less strongly ITGA2, ITGB6 and MMP1 compared to untreated BM-MSC (Fig. 3B, red circles). Exosome treatment also triggered a decrease in AGT and BCL2. Those transcripts that could discriminate exosomes from sTGFβ stimulation are shown (Fig. 3B, right, green circles), and include HGF, IL5, CCL2, TGFB3 in addition to the metalloproteinase's. Whilst there was an elevation in mRNA for VEGFA with exosome stimulation, the changes were below our chosen threshold for consideration as differentially expressed (<3x fold change). In independent experiments, qRT-PCR was used to confirm exosome mediated changes in the transcripts demonstrating particularly strong (160-fold) elevation in MMP-3 and also elevated MMP-1, MMP-13, and SerpinA1, with decreased mRNA for AGT, showing broad agreement with the array data (Fig. 3C). The levels of these transcripts present in RNA isolated from DU145-exosomes were negligible, and therefore do not account for the observed changes (Fig. S3). Together the array shows that exosomes impart a phenotype that has some overlap with that of sTGFβ stimulus, but points to some unique features including heightened HGF and matrix regulating proteases such as MMP's.

### Exosome generated myofibroblasts exhibit pro-angiogenic functions

We next explored some possible functional aspects of exosome-differentiated BM-MSC, examining whether or not exosome-mediated differentiation gives pro-angiogenic function. To do this we investigated several aspects of endothelial cell behaviour *in vitro*.

**Figure 4:**
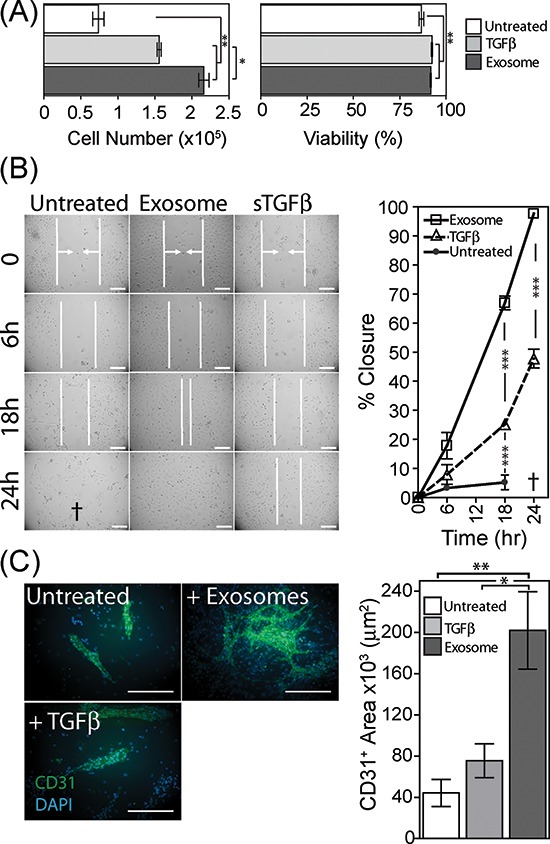
Pro-angiogenic functions of exosome-differentiated BM-MSC Conditioned media from BM-MSC pre-treated for 4 days as specified, was added to wells containing 1 × 10^4^ primary HUVEC. Following 6 days in culture, HUVEC were harvested and viability and cell counts performed using the ViaCount system on a Guava flow cytometer (Bars, mean ± SD, of triplicates) **(A)**. Confluent monolayers of HUVEC were established, prior to scrapping a single vertical scratch using a 200μl pipette tip. CM from BM-MSC pre-treated as specified, were added, and the distance between two sides of the scratch, highlighted by vertical white lines and arrows, was monitored at specified time points microscopically up to 24 h. The symbol † depicts a loss of HUVEC adhesion at 24 h, hence scratch width could not be measured. (Graph shows Mean ± SD, of duplicate wells per treatment. Data are representative of two such experiments **(B)**. Monolayers of BM-MSC were pre-treated as specified for 4 days, at which point 50% of the culture medium was removed, and replaced by the same volume of EBM2 endothelial cell culture medium lacking growth factors, containing 20,000 HUVEC per well. After further 6 day incubation, the co-cultures were fixed and immune-fluorescently stained for CD31 (green) and DAPI (blue) (Scale, 100μm). Quantification of surface area of the CD31-positive structures was performed (Bars, Mean ± SD of triplicate wells per condition). Representative of three such experiments **(C)**.

Firstly we examined the proliferation and survival properties of umbilical vein-derived endothelial cells (HUVEC) in the presence of BM-MSC conditioned medium (CM), collected from BM-MSC pre-treated for 4d with exosomes or sTGFβ. The CM was added in the absence of exogenous endothelial-cell growth factors, and at day 6, cell viability and cell numbers were determined by flow cytometry. The viability of endothelial cells grown in BM-MSC conditioned medium was >75%, but there was a small increase using CM from either sTGFβ or exosome-treated BM-MSC. The endothelial cell numbers expanded poorly with CM from untreated BM-MSC, and there was a weak proliferative response to CM from sTGFβ treated BM-MSC. This was slightly stronger, however following exosome treatment of BM-MSC and lead to a 3-fold elevation of endothelial cell numbers at day 6. Exosome-differentiated BM-MSC, therefore, produce factors which support endothelial cell expansion (Fig. 4A).

We also examined the influence of BM-MSC on endothelial cell migration using an endothelial monolayer scratch assay. Conditioned media, from exosome-treated BM-MSC, accelerated scratch closure, with full closure of the scratch occurring by 24 h. In contrast the scratch exposed to sTGFβ-treated BM-MSC conditioned medium was only 50% closed by 24 h (Fig. 4b). At this time point, the endothelial cells were alive but poorly adherent in the presence of CM from untreated BM-MSC, and as such it was not possible to determine the position of the scratch margins at 24 h. Nevertheless, scratch closure for this treatment was clearly less complete at 6 and 18 hr compared to the other treatments (Fig. 4B), and this phenomenon was apparent in three independent experiments.

Lastly, we performed an *in vitro* tubule-formation assay as a measure of proliferation, migration and cell organisation, through co-culture of pre-treated BM-MSC monolayers with endothelial cells, as described [37]. BM-MSC were treated with exosomes or sTGFβ for 4 days prior to the drop-wise and scattered addition of endothelial cells to the wells. After a further 6 days cells were fixed and stained for the endothelial marker CD31. In wells containing untreated BM-MSC some clusters of CD31-positive cells formed on top of the BM-MSC monolayer, but these were relatively rare, forming short structures with no evidence of branching. In contrast, exosome-treatment of BM-MSC allowed the support of multiple branched, long and wide structures consistent with supporting more elaborate vessel-like structures [38]. There was a significant elevation in the total surface area occupied by CD31-positive cells (Fig. 4C). Stimulating BM-MSC with sTGFβ failed to generate such structures, instead generated structures similar to those seen for untreated BM-MSC. In summary, exosome-differentiated BM-MSC support the proliferation, motility and organisation of endothelial cells and are consistent with a pro-angiogenic function.

### Exosome generated myofibroblasts support tumour cell expansion, migration and invasion

Cancer associated stroma may also be considered as a direct stimulus for tumour growth; we therefore examined the impact of exosome-generated myofibroblasts on Du145 prostate cancer cells.

**Figure 5:**
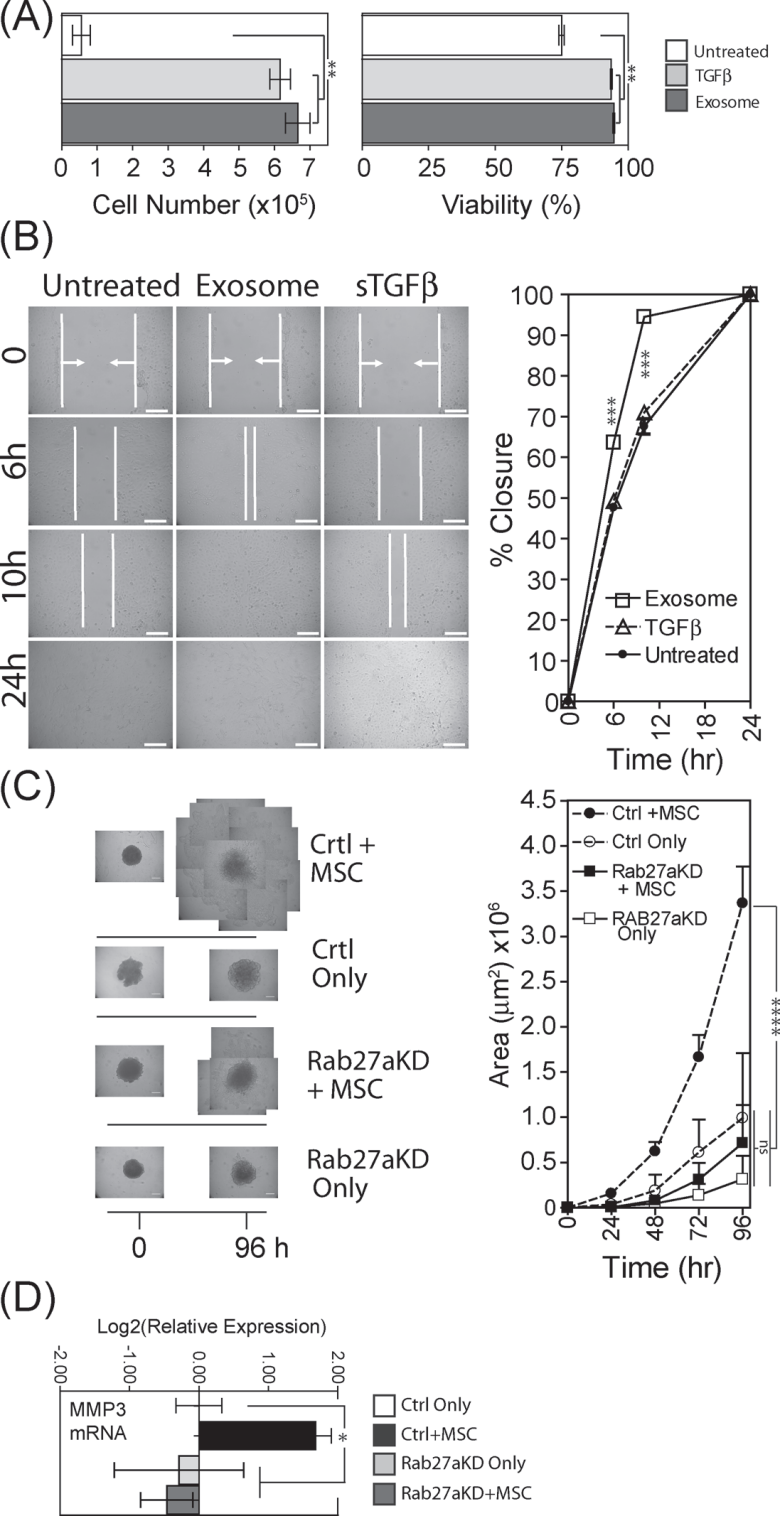
Tumour-growth promoting functions of exosome-differentiated BM-MSC Conditioned media from BM-MSC pre-treated for 4 days as specified, was added to wells containing 1×10^4^ DU145 tumour cells that had been grown under serum-free conditions for 24 h previously. Following 3 days in culture, Du145 cells were harvested and viability and cell counts performed using the ViaCount system on a Guava flow cytometer (Bars, mean ± SD, of triplicates) **(A)**. Confluent monolayers of Du145 tumour cells were subject to a single vertical scratch, and CM from pre-treated BM-MSC was added. The distance between the scratch margins was measured at time points up to 24 h. (Bars, Mean ± SD, duplicate wells) **(B)**. Spheroid cultures were established in poly-haema coated plates, composed of Du145 cells (control or Rab27^KD^), or a combination of Du145 cells with BM-MSC (at a ratio of 4:1 respectively), at 10^4^ total cells/spheroid. After 4 days when cells had formed firm spheroid structures, they were transferred to fresh wells and Matrigel™ was added. The area occupied by extra-spheroidal cell outgrowths was measured daily for up to 96 hr. For late time-points, multiple images of the spheroid-outgrowths were taken and these were tiled to form a composite representation of the full extent of outgrowth. (Graph shows Mean ± SD, quadruplicate spheroids per treatment) **(C)**. In order to obtain enough RNA, 5 such spheroids were pooled) and total RNA was extracted and evaluated for levels of MMP-3 mRNA. Columns represent Log_2_(relative expression) ±SD, compared to untreated control tumour only (based on technical triplicates) **(D)**.

CM was taken from 4 day differentially treated BM-MSC and added to tumour cells for 3 days prior to assessment of cell number and viability by flow cytometry. CM taken from either sTGFβ or exosomes stimulated BM-MSC exhibited a pro-proliferative effect, with cell number increased 6 to 7 fold after 3 days, compared to CM from untreated BM-MSC (Fig. 5A). There was also an increase in cell viability with these treatments, consistent therefore with direct MSC-support of tumour cell expansion. These effects were not due to carry-over of cancer exosomes within the MSC CM as these controls showed no such activity (not shown) pointing to MSC-derived factors as essential.

We also used a monolayer scratch assay, to examine tumour cell motility in response to the same CM. Scratch closure following treatment with CM from sTGFβ-BM-MSC was not different from untreated control, with closure at ~70% after 10 hr. There was, however, a significant (*p* < 0.001) increase in scratch closure when using CM from exosome treated BM-MSC, with 95% closure at the same time point (Fig. 5B). Exosome-treated BM-MSC therefore produce factors that enhance tumour cell motility.

We also established 3D-spheroid cultures, incorporating either tumour cells alone or tumour cells with BM-MSC (at a ratio of 4 tumour cells to 1 BM-MSC), in order to mimic an *in vivo* 3D microenvironment. In these experiments we used the aforementioned exosome deficient tumour cells (Rab27a^KD^), and the respective vector control cells. After establishing spheroids for four days, spheroids were transferred to fresh wells and Matrigel™ was added burying the spheroids in a 3D basement-membrane mimic. Each well was microscopically examined for 96 hours, to ascertain whether or not there was any effect on escape of cells out from the spheroid into the surrounding matrix. Control or Rab27a^KD^ tumour cells alone showed a paucity of cell outgrowth even at 96 hours (Fig. 5C). In marked contrast, combining BM-MSC with control tumour cells revealed notable outgrowth as early as 24 hours (Fig. S3), and growing beyond the field of view of the objective at 48 hours. By tiling multiple images we were able to continue to assess invasion for up to 96 hours demonstrating a highly significant increase in the area occupied by extra-spheroidal cells (*p* < 0.0001) (Fig. 5C, graph). In contrast, exosome deficient tumour cells together with BM-MSC invaded the matrix somewhat later at 48 hours (Fig. S4), and this was drastically less extensive by 96 hours (Fig. 5C), with a clear attenuation of invasive capacity in the absence of an intact exosome secretion pathway. As MMP-3 was highly elevated (>100 fold) in BM-MSC in the presence of exosomes (Fig. 3C) we extracted RNA from a pool of 5 spheroids per treatment group and examined possible alterations in this transcript within the spheroids. The same pattern was apparent here also, with heightened MMP-3 mRNA found only in the presence of BM-MSC together with control tumour cells (Fig. 5D). Omission of BM-MSC or using Rab27a silenced tumour cells did not show MMP-3 elevation. Therefore exosome-mediated MMP-3 induction in BM-MSC correlates with a pro-invasive function.

In summary, exosome-differentiated BM-MSC directly promote tumour proliferation and motility. Assistance from BM-MSC is required to trigger the matrix invasion characteristics of the 3D-spheroid model, and inhibiting exosome secretion here strongly attenuates tumour invasion.

## DISCUSSION

The cross-talk between cancer cells and local stromal cells has seen renewed interest in recent years, with mounting evidence for a profound influence of fibroblastic stroma in driving disease progression and poor clinical outcome [8, 39]. Several studies have identified common characteristics of cancer associated stromal cells across diverse solid cancer types. These include molecular traits of myofibroblasts, which promote tumour cell growth directly and stimulate angiogenesis [11, 40–41]. However, some recent reports in pancreatic carcinoma where myofibroblasts are conditionally ablated by genetic means at different time points during carcinogenesis, reveal heightened angiogenesis and immune-suppression following removal of myofibroblasts, with worsening overall survival of mice [42–43]. Such studies suggest that in this model at least, stromal myofibroblasts play a protective role in attenuating the disease [43]. Whilst currently such data is lacking for other cancer types, the prevailing paradigm views cancer-associated myofibroblasts as prognostically poor. There remains however, incomplete understanding about stroma, its cellular and molecular nature and its consequence in terms of disease outcome.

Precisely how myofibroblastic traits of cancer associated stroma are initiated; altering stroma from a normal homeostatic function towards a malevolent or indeed a protective phenotype remains unclear. Although TGFβ secreted by cancer cells has remained among the principal culprits driving myofibroblastic differentiation, studies by us [28–29] and others [30–32, 44] have recently highlighted a role for exosome vesicles in delivering TGFβ to elicit stromal modulation in cancer. Local tissue fibroblasts exhibit the capacity to differentiate into myofibroblasts under the influence of soluble TGFβ, and this requires additional factors such as endogenous hyaluronic acid production and the interaction between CD44 and the EGF receptor [45]. The detailed myofibroblastic phenotype arising however appears to differ from cancer associated stromal cells, as it is not pro-angiogenic, and fails to enhance tumour growth in xenografts [29]. In contrast, stimulating the same stromal cell source with TGFβ-bearing prostate cancer exosomes generates myofibroblasts that mimic those extracted from cancerous tissues; driving angiogenesis and *in vivo* tumour promotion [29]. The onset of αSMA stress fibres in stromal cells is not therefore directly coupled to their tumour-modulating function; and a better surrogate indicator of stromal functionality may be the secretion of certain factors including SDF-1 [24], VEGF and HGF [29].

In our presented study, TGFβ-bearing prostate cancer exosomes modulated the differentiation of BM-MSC, giving rise to a myofibroblastic phenotype exhibiting heightened VEGF and HGF secretion. The cell arising, therefore represent those aforementioned traits of cancer associated stroma even though the originating cell source is distinct. This suggests that it is the nature of the trigger (i.e. exosomes), rather than the originating cell type, that is most important for the function of the myofibroblast arising. In contrast, soluble TGFβ stimulation of BM-MSC gave a radically different response that lacked onset of αSMA-stress fibres or elevated VEGF, and reduced constitutive HGF secretion. With different sources of both cancer exosomes and MSC, other studies have also described exosome-mediated myofibroblastic differentiation of adipose tissue or umbilical cord blood-derived MSC [30–31, 44] and show the role of TGFβ, and SMAD-dependent signalling in this process. Comparing directly, however, the effects of exosomes and sTGFβ, as we have done highlights the profound difference in the cell response arising, with the exosomal-form of TGFβ giving a clearly distinctive molecular and functional phenotype. A detailed mechanistic explanation for this difference is currently lacking, and given the molecular complexity of exosomes secreted by cancer cells, represents a significant challenge to define. Certainly for fibroblast stimulation by exosomes, we revealed that intact heparan sulphate proteoglycans at the exosome surface were required for functional delivery of vesicular TGFβ [29]. The possible co-delivery of other factors such as growth factors, or vesicular mRNA or miRNA are aspects we are currently examining. What is clear is the exosome-mediated generation of myofibroblasts from BM-MSC is a TGFβ-dependent process which produces a phenotype with the expected characteristics of cancer associated stromal cells.

We have not yet addressed the nature of the MSC response to exosome stimulation in terms of the cell population, and are currently unclear as to whether the response is homogenous, or whether it is a sub-population of MSC which differentiate into myofibroblasts that subsequently proliferate to take over the population. We present evidence that around 60–70% of MSC exhibit αSMA positivity after around 2 weeks which is quite different from stimulating fibroblasts where essentially ~100% of cells become positive by 3 days [28]. Such observations suggest a more heterogeneous response with MSC as a stromal cell source. The question may have *in vivo* relevance, as the infiltration of the cancer microenvironment by few MSC may be sufficient to generate an expanding population of myofibroblastic cells in situ. We are currently addressing such questions to gain greater insight into such exosome-mediated changes in subpopulations of stem cells.

The functional properties of the exosome-generated myofibroblasts support the premise that cancer exosomes have a disease-promoting influence. Although the direct impact of cancer exosomes on angiogenesis has been well documented [37, 46], the impact of exosome-modified BM-MSC on this process has not to our knowledge been studied. Endothelial cells exhibited preferential proliferation and migration in the presence of soluble factors produced by exosome-differentiated BM-MSC. We also document an enhanced capacity to form complex vessel-like structures. These were akin to structures produced using natural, cancer-tissue derived stromal cells of prostate origin [29]. In a similar fashion, we show a direct positive effect of exosome-generated myofibroblasts on tumour cell proliferation and motility, but moreover a heightened propensity to invade into extracellular matrix using a 3D spheroid model. Extensive invasion was abrogated when targeting exosome secretion by Rab27a silencing, and invasion was absent when BM-MSC were left out of the spheroids highlighting the role of this stromal cell type in dictating invasive behaviour. We do not know whether the invading cells are principally epithelial or mesenchymal in nature, but given the predominance of tumour cells (4:1) in the spheroids, and the pro-proliferative influence of BM-MSC on tumour cells, the invading cells are most likely to be principally epithelial as their morphology would suggest. This high invasive capacity of the system agrees with additional evidence showing exosome-driven metalloproteinase elevation in BM-MSC, an aspect peculiar to exosome-stimulus and not found when using sTGFβ1. Such factors, which include the collagenases MMP-1 and MMP-13 and the stromelysin MMP-3, have well documented roles in disease progression and can in particular aid cell penetration through extracellular matrices, supporting invasion and metastasis in several cancer types [47]. Notably a recent study highlighted bone-marrow derived myofibroblasts found at the primary tumour site in a skin cancer model as the principal source of MMP-13 in situ [48], and that this MMP was required for subsequent invasive behaviour [48–49]. Furthermore, a recent study has demonstrated that down-regulation of MMP-3 in cancer-associated fibroblasts subsequently attenuated prostate cancer cell invasion [50]. In addition a novel role for proteases in association with stromal-cell derived exosomes appears to be an important element in controlling stromal cell phenotype and subsequent microenvironmental changes. Stromal exosomes taken from cells deficient in TIMPs, harbour bioactive enzymes such as ADAM10 that support the generation of myofibroblasts with classical traits of tumour promotion including heightened HGF, SDF1 and other factors [51]. Whether exosomes of differentiated MSC exhibit such proteolytic activities and functions are not currently known. Other transcripts modulated by exosomes were ITGB6 and ITGB8 encoding for components of the integrin αvβ6 and αvβ8 respectively, which are implicated in the conversion of latent-TGFβ to bioactive TGFβ in several systems [52–53]. The importance of these exosome-mediated integrin changes in BM-MSC for TGFβ activation and adhesive functions has not yet been investigated but certainly deserves future attention. The functional data in relation to spheroids and to the pro-angiogenic properties of differentiated BM-MSC correlate well with the phenotypic alterations induced by exosome stimulation. These changes emphasise the profound role of MSC-cooperation with tumour cells in driving distinct aspects of disease progression, and the requirement for a functional cancer exosome secretion pathway for this influence to be fully realised.

We acknowledge that the specificity of the Rab27a silencing approach for inhibiting the secretion of exosomes and not other factors is somewhat controversial [54], and we are cautions about over reliance on this as a sole means of showing a role for exosomes. Soluble cancer-cell derived factors have been shown through prolonged exposure to mediate myofibroblastic differentiation of MSC [24], and we clarify through some simple experiments that exosomes are the dominant factor in this process. Reducing the level of exosomes in cancer cell conditioned media, by high speed centrifugation, generated cell conditioned media that was poor in driving myofibroblast differentiation. The activity resides in the 120,000xg pelletable fraction, strongly implicating exosomes as mechanistically central to cancer-mediated control of MSC. In addition, the use of purified exosomes and the Rab27a silencing approach collectively provide data supporting these conclusions.

MSC exhibit enormous cellular plasticity, capable of a broad range of differentiation programmes. These are complex to demonstrate *in vitro* however, requiring assorted hormonal or growth factor stimulations applied sequentially often over long time periods. For tissue engineering applications, optimising and simplifying such protocols remains a major biotechnological challenge in the context of cellular therapeutics. Here we have only explored adipogenic differentiation as one of the classical differentiation routes for BM-MSC. The addition of TGFβ-positive exosomes during this form of differentiation was sufficient to block and fully override the formation of adipocytes to preferentially generate myofibroblasts. What is equally remarkable is that a single stimulation with exosomes was sufficient to trigger the onset of αSMA-positive cells over the course of 2 weeks. This opens novel exploratory avenues for manipulating MSC differentiation using various exosome sources to dictate the desired differentiation programme. Although supporting evidence is currently lacking, our data suggest cancer exosomes may be capable of overriding the natural control of MSC differentiation *in vivo*, away from a self-renewal or reparative phenotype towards undesirable disease promoting myofibroblasts.

We do not currently know if such exosomes are capable of distant communication from the primary site of prostate, to bone marrow. In other systems, melanoma exosomes have documented roles in mobilising bone-marrow haematopoietic and non- haematopoietic progenitors which in turn influence disease progression [55]. The role of prostate cancer exosomes in mobilising BM-MSC remains an open question of considerable interest.

In conclusion, our report identifies prostate cancer exosomes as potent factors for controlling the phenotypic and functional differentiation of BM-MSC towards a pro-angiogenic and pro-invasive myofibroblast. The phenotype is similar to that reported for cancer associated stromal cells, with exosomes and not other soluble factors required to generate this dominant form of differentiation. We therefore advocate that molecular targeting of this exosome-driven process in a clinical setting is likely to attenuate tumour-manipulation of the local microenvironment, and slow disease progression.

## MATERIALS AND METHODS

### Cell culture

The prostate cancer Du145 [56] cell line was purchased from ATCC, and used for all the experiments shown. Some confirmatory experiments in relation to exosome driven MSC-differentiation were also performed using the PC3 prostate cancer cell line (also from ATCC), with similar effects (not shown). The cells were seeded into bioreactor flasks (Integra, Nottingham, UK), and maintained at high density culture for exosome production, as previously described [57]. The cells were cultured in RPMI 1640 (Lonza, Wokingham, UK), supplemented with penicillin/streptomycin and 10% foetal bovine serum (FBS; Life Technologies, Paisley, UK). FBS was depleted of bovine exosomes by overnight ultracentrifugation at 100,000 g, followed by filtration through 0.2 μm and then 0.1 μm vacuum filters, (Millipore, Hertfordshire, UK). For some experiments Du145 rendered deficient in Rab27a using a ribozyme knockdown method, were used [29]. Human bone marrow MSC were purchased from Promocell (Heidelberg, Germany). And expanded according to the suppliers instructions using Promocell culture media, with supplement mixture. For MSC-differentiation experiments the culture medium was DMEM-low glucose (1 g/l) (Lonza) with 10% MSC-optimised FBS (also rendered exosome depleted as above). All experiments were conducted with early passage MSC (up to passage 5). Cells were confirmed negative for mycoplasma contamination by monthly screening (MycoAlert; Lonza). Adult lung fibroblasts (Coriell Institute for Medical Research, USA) were maintained in DMEM/F12 (Lonza) and 10% exosome depleted FBS. Human umbilical vein endothelial cells (HUVEC) were purchased from Lonza, and maintained using the EBM2-bullet kit. For functional assays, these additional growth factor supplements were withdrawn for the duration of the experiments.

### Exosome purification

Prostate cancer cell conditioned media (CM) was subjected to serial centrifugation to remove cells (400 g, 10 min) and cellular debris (2000 g, 15 min). The supernatant was then filtered (0.22 μm), to remove remaining debris and larger vesicles. The clarified CM was under laid with a 4ml cushion of 30% Sucrose/D2O, and following 2 h ultracentrifugation at 100,000g (in a SW32 rotor, Beckman Coulter), the cushion was collected, and washed in excess PBS by ultracentrifugation. The pellet was resuspended in 50–100μl PBS and frozen in aliquots at −80°C. Unless stated otherwise, purified exosomes were used in assays at a dose of 150 μg/ml which is equivalent to a TGFβ dose of 1ng/ml [34]. Protein concentrations were evaluated using a microBCA protein assay (Pierce/Thermo, Northumberland, UK), and specified exosome doses used in experiments are based on this. The nano-particles in each preparation were quantified by nanoparticle tracking analysis (Nanosight, Malvern Instruments, Amesbury, UK) as described [58]. Preparations used for the study exhibited a particle : protein ratio of 2 × 10^10^ or greater, and were therefore considered high purity [58], and satisfy the size, density, structure and molecular phenotype of exosome vesicles as described [29]. To deplete exosomes from CM, samples were ultracentrifuged for 1.5 hr at 120,000g (in a TLA110 rotor, Optima-Max Ultracentrfuge; Beckman Coulter, High Wycombe, UK), after which the supernatants contain ~90% fewer nano-particles measured by nanoparticle tracking analysis (Nanosight, Malvern Instruments, Amesbury, UK) (not shown).

### Flow cytometry

BM-MSC, fibroblasts or myofibroblasts were harvested using acutase, and incubated on ice for 1 h with directly conjugated antibodies including; SSEA-4-FITC (R&D Systems), CD44-PE (BD Bioscience), CD90-PE, CD105-APC, CD14-APC, CD45-PECy5 (eBioscience, Hatfield, UK), CD73-PE (BD Biosciences, Oxford, UK). Indirect staining was performed for GD2 (BD Biosciences), with a secondary goat anti-mouse-Alexa488 conjugated antibody at 1:200 for 40 min (Life Technologies). Cells were analysed using a FACScanto cytometer (Beckton Dickinson, Oxford, UK), and data analysed using FACS Diva (v6.2).

### Adipogenic differentiation

BM-MSC or lung fibroblasts were cultured in 24 well plates and once confluent were given adipogenic induction medium (DMEM containing insulin, dexamethasone, indomycine and IBMX) as described [59]. In addition soluble recombinant human TGFβ1 (1ng/ml) or DU145 exosomes (150 μg/ml) was added along with the induction medium to some wells. Fresh adipogenic induction medium was given every 2–3 days over a period of 21 days, with the exception of day 7 and day 15, in which maintenance medium (DMEM with only insulin and FBS) was given. After 21 days of differentiation, adipocytes were fixed in 4% (w/v) paraformaldehyde and lipid droplets stained with Oil Red O Solution and counterstained with haematoxylin solution (Millipore).

### Immuno-fluorescence microscopy

BM-MSC were cultured in growth factor free conditions for 24 h hours before stimulation with exosomes or sTGFβ at specified doses and times. In some experiments this was done in the presence of a neutralising TGFβ-antibody at 10 μg/ml (R&D Systems, Abingdon, UK), or an inhibitor of the Alk-5 TGFβ-receptor-I (SB43152) at 10 μm (Sigma-Aldrich, Dorset, UK). Cells were gently washed 3x in pre-warmed PBS. Cells were fixed in fresh, ice-cold acetone:methanol (1:1 v/v), for 5 min and allowed to completely dry in air at room temperature. Cells were blocked for 1.5 h in 1% BSA/PBS (w/v) protease free (R&D Systems). Anti α-smooth muscle actin (Santa Cruz Biotecnology, Santa Cruz, CA, USA) was used at 1 μg/ml (in 0.1% BSA/PBS w/v) for 1 h, and counterstained with DAPI for 10 min before washing 4 times in PBS. Cells were visualised by wide-field fluorescence (AxioVert, Zeiss, Cambridge, UK), and the proportion of αSMA-positive cells was manually counted across 6 microscopic fields, and triplicate treatments unless stated otherwise.

### ELISA

The quantity of VEGF-A or HGF present in the cell conditioned media of BM-MSC was assayed using the DuoSet ELISA system (R&D Systems) according to manufacturer's instructions, except for the detection stage where the coluorimetric HRP-based detection was substituted for streptavidin-conjugated Europium (PerkinElmer, Cambridge, UK), and time resolved fluorimetry performed on a Wallac Victor2 (PerkinElmer).

### PCR-arrays

To identify potential differences in the phenotype of differentiated MCS following exosome or sTGFβ treatment, cellular RNA was extracted at day 3 using Tri-Reagent (Sigma-Aldrich) as per the manufacturer's protocol. Total RNA (0.5 μg) was reverse transcribed (RT^2^ First Strand Kit; Qiagen, Manchester, UK), prior to amplification using a low density PCR array (RT^2^ Profiler PCR Array; Qiagen) covering 84 transcripts of known association with fibrosis. This was completed in accordance to the manufacturer's instructions, and performed as biological triplicates for each treatment condition (9 arrays in total). The comparative Ct method was used for relative transcript quantification against the average ΔCt derived from internal controls (β-actin, β-2-microglobulin, GAPDH, HPRT1, and RPLP0). Data were analysed using the supplied software, and presented as volcano plots with a *p*-value threshold of <0.05 and a fold-change threshold of ±3. Selected transcripts were verified using TaqMan PCR gene expression assays (Life Technologies), as described [28]. Target gene expression was quantified, using the comparative Ct method, relative to that of a standard reference gene (GAPDH). All PCR amplifications were performed in a StepOne Plus Real-Time PCR System thermocycler (Life Technologies).

### Functional assays

Prior to functional assessments, endothelial cells or Du145 tumour cells were cultured for 24 h in growth-factor free conditions prior to stimulations. BM-MSC were pre-treated for 4d with exosomes (150 μg/ml) or sTGFβ1 (1ng/ml) and conditioned media was harvested. BM-MSC was added to endothelial cells at a ratio of 1:1 (v/v) with EBM2-medium and incubated for 6 days. Endothelial cells were harvested using accutase, and cell number and viability measured using ViaCount stain and Guava EasyCyte flow cytometer (Millipore). For Du145 tumour cells, BM-MSC CM was added at 1:1 ratio (v/v) with RPMI media for 3 days prior to measuring cell number and viability as above. These measurements were performed in triplicates. To assess cell motility, a confluent monolayer of endothelial or tumour cells in 24 well plates, were subject to a single vertical scratch, using a 200μl pipette tip. BM-MSC CM was added as above, and wells were microscopically monitored up to 24 hours. The width of the scratch in duplicate wells was measured at 4 points for each well, using Image-J (National Institutes of Health, Bethesda, MD, USA), and the rate of monolayer recovery plotted as relative to the original scratch width (% closure) as described [29]. Formation of endothelial tubules was performed as described [29], with endothelial cells (20,000/well) added in triplicate to monolayers of BM-MSC that had been previously treated with exosomes or TGFβ for 4days. After a further 6 days culture, structures formed by endothelial cells were visualised by immune-fluorescent labelling of CD31 (SantaCruz). The total area occupied by CD31-positive structures was quantified using the free-hand selection tool in Image-J to calculate the area occupied by stained cells in each well. Data show the average from triplicate wells per treatment, and are representative of three such experiments.

### Tumour cell and BM-MSC heterotypic spheroids

Tumour cells (control or Rab27a^KD^) were incubated alone or together with BM-MSC at a ratio of 4 tumour cells:1 MSC, in Poly(2-hydroxyethyl methacrylate) haema (Sigma, Dorset, UK) coated 96-well, “u”-bottom plates. The medium used consisted of 1:1 ratio (v/v) of RPMI and DMEM (low glucose) in 10% FBS. The total number of cells was 10^4^ per well. After 4 days, the cells had established 3D-spheroidal structures. There was no significant difference in the size or structure of the spheroids at these time points (not shown). To evaluate potential changes in invasive behaviour of the cells, spheroids were transferred to fresh uncoated 96 well plates and Matrigel™ (Corning, Flintshire, UK) was added (100μl/well). After setting at 37°C for 30 min, medium was added, and the wells monitored microscopically for 4 days thereafter. To estimate the magnitude of invasion out from the spheroid, the free-hand selection tool in Image-J was used draw the circumference of the central sphere. This was subtracted from the circumference of the region occupied by invading cells. This gives an approximation of the area of Matrigel™ invaded by cells, as it does not take account of the volume aspect of the 3D culture, and is likely therefore to underestimate the true differences across the treatments.

### Statistical analysis

Statistic analyses were performed using Prism-4 software V4.03 (Graph Pad, San Diego, CA, USA). In experiments with more than two experimental groups, one-way ANOVA with Tukey's post-test was used, except for migration and *in vivo* experiments where a two-way ANOVA with Bonferroni post-test was used. Experiments with two experimental groups were evaluated using Student's t-test. P-values less than 0.05 are considered significant *P < 0.05, **P < 0.01, ***P < 0.001, ****P < 0.0001.

## SUPPLEMENTARY FIGURES AND TABLE


