# Supplementary material for: Cancer exosomes trigger mesenchymal stem cell differentiation into pro-angiogenic and pro-invasive myofibroblasts
Source: Oncotarget. 2014 Nov 28;6(2):715–31. doi: 10.18632/oncotarget.2711 (PMC4359250; doi:10.18632/oncotarget.2711)
Supplement: Supplementary file 1 [file oncotarget-06-715-s001.pdf]

SUPPLEMENTARY FIGURES AND TABLE

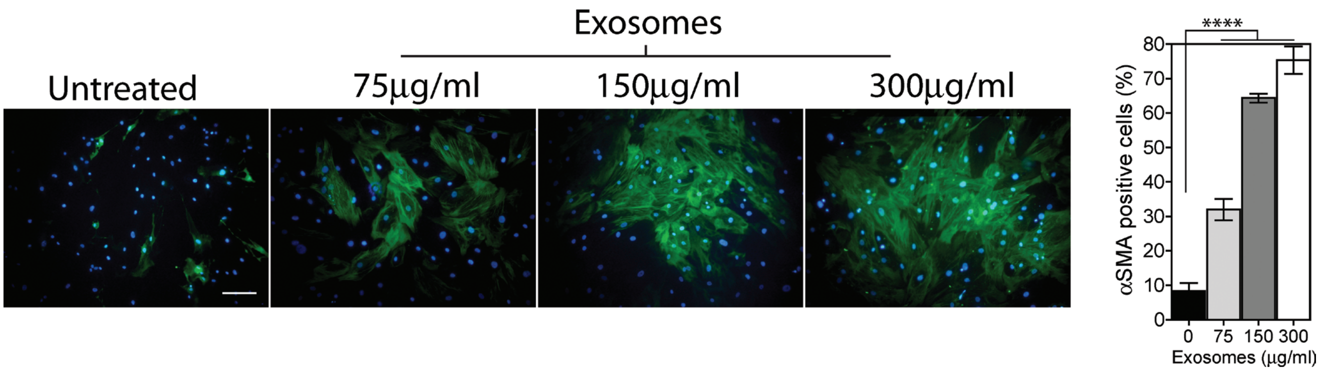

Supplementary Figure S1: Exosome dose response. M-MSC were treated with Du145 exosomes (0-300 µg/ml) and after 14 day incubation, cells were fixed and stained for αSMA (green) and DAPI (blue) (Scale, 100 µm). Bars, also presented as Fig. 2C show the mean (± SD) proportion of αSMA positive cells per field of view, from a total of 6 microscopic fields examined in duplicate wells per treatment**.**

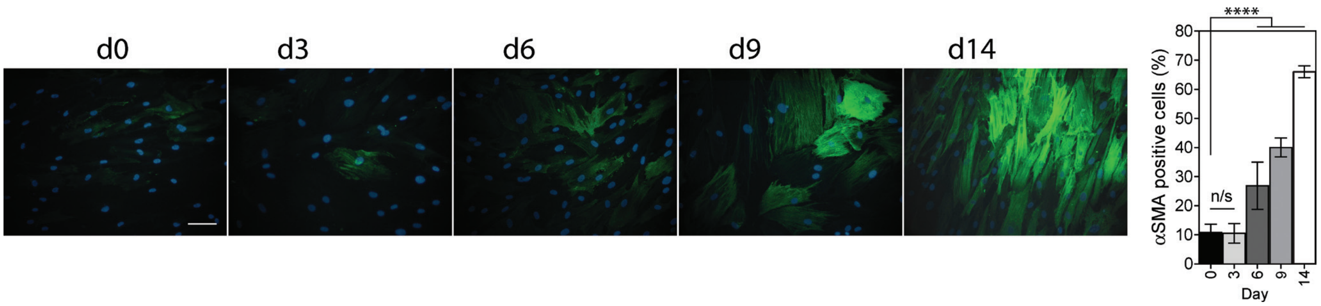

Supplementary Figure S2: Kinetics of exosome-mediated MSC differentiation. BM-MSC were treated with Du145 exosomes (at 150 µg/ml) and at indicated periods up to 14 days cells were fixed and stained for αSMA (green) and DAPI (blue) (Scale, 100 µm). Bars, also presented as Fig. 2D show the mean (± SD) proportion of αSMA positive cells per field of view, from a total of 6 microscopic fields examined in duplicate wells per treatment.

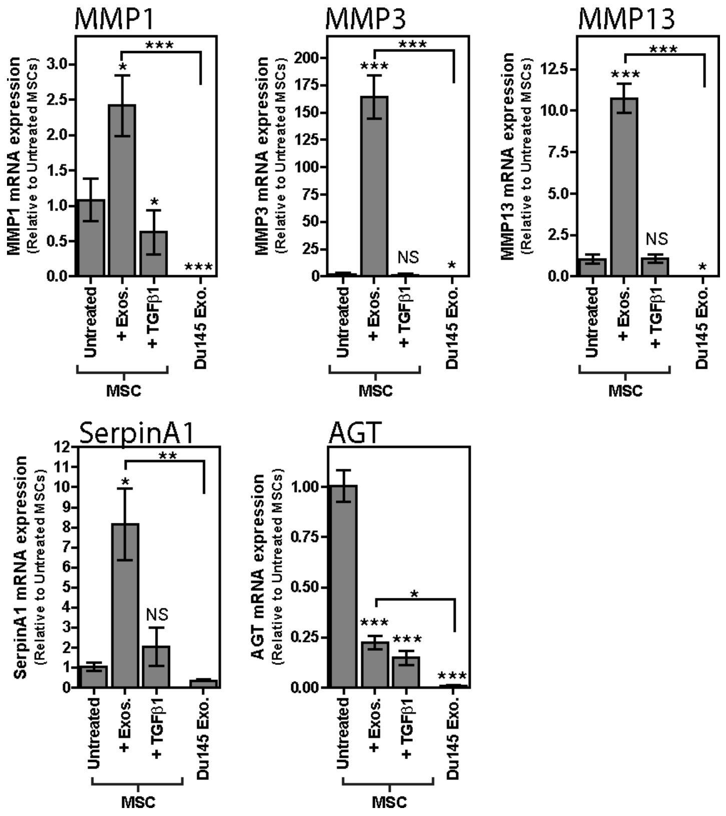

Supplementary Figure S3: Exosomal mRNA does not account for changes in the MSC-transcriptome. Total RNA (0.5 µg), isolated from DU145 exosomes was reverse transcribed and analysed by TaqMan PCR gene expression assay for the specified transcripts. This was compared to the same amount of total RNA extracted from MSC stimulated with exosomes or TGFβ or left untreated as specified. This reveals the principal origin of these transcripts in exosome-activated MSC is of MSC and not exosome origin.

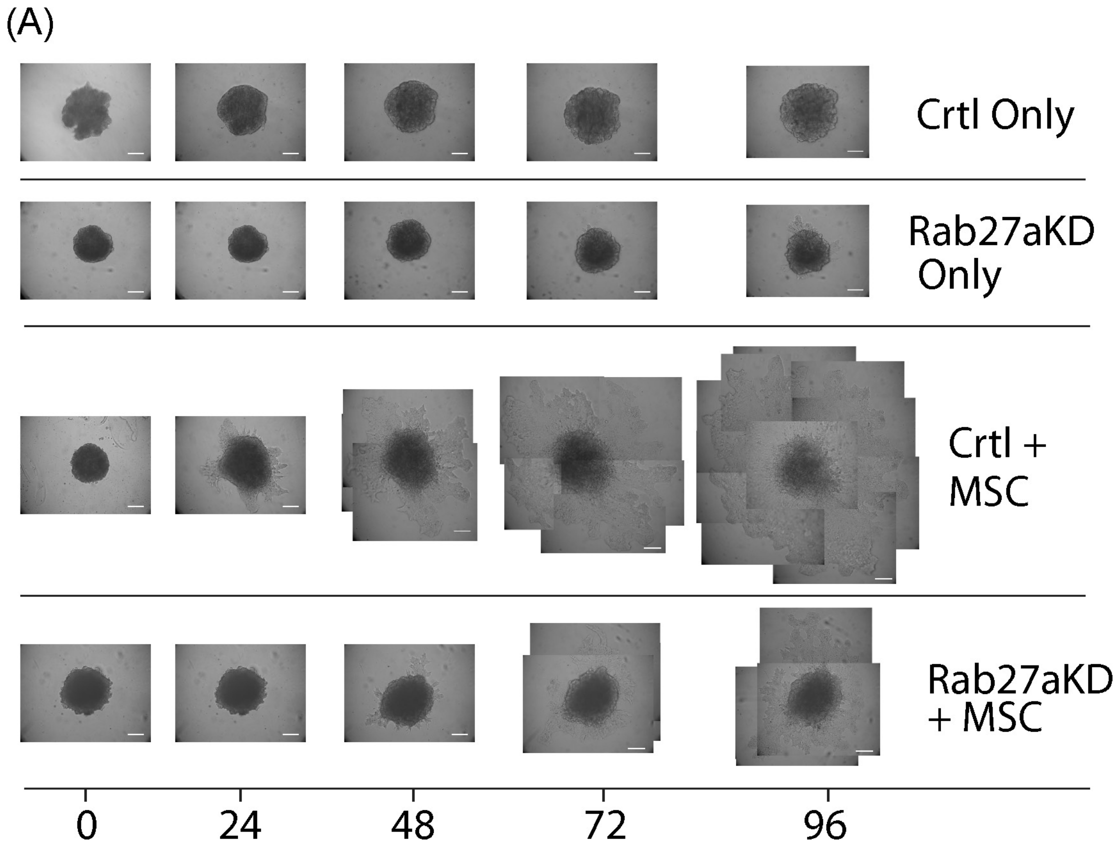

Supplementary Figure S4: Spheroid invasion assay time-course. 3D Spheroids were established using tumour cells alone (control Du145 or Rab27a-KD Du145 cells) or together with BM-MSC (at a ratio of 4 tumour cells to 1 BM-MSC), with a total of 1 x 10^4 cells/spheroid. After 4 days, spheroids were transferred into fresh 96-well plates and Matrigel™ was added. The outgrowth of cells from the sphere was microscopically monitored at specified times up to 96 hours. Each condition had quadruplicate spheroids, and a single representative sphere from each condition is shown. Where the outgrowth extended beyond the visual field of the 10x objective used, multiple images were taken and these have been tiled to allow the full extent of outgrowth to be assessed.

Supplementary Table S1: Raw data from the PCR-profiler array, depicting the mean fold change (from triplicates) and *p* value across the specified treatments for the array coverage of 83 transcripts. Those highlighted in bolded text signify changes considered differentially expressed, according to the criteria of +3-fold change compared to un-stimulated BM-MSC and a *P* value <0.05

	**Exosomes vs Untreated**		**TGFbeta vs untreated**		**Exosomes vs TGF-beta**	
mRNA	FOLD Change	*p* Value	FOLD Change	*p* Value	FOLD Change	*p* Value
ACTA2	0.9834	0.9930	1.9776	0.0123	0.4973	0.0183
AGT	**0.2326**	**0.0318**	0.4236	0.0572	0.5492	0.1434
AKT1	0.8463	0.2831	0.7163	0.2052	1.1815	0.5812
BCL2	0.3112	0.0042	0.3901	0.0003	0.7978	0.6274
BMP7	1.0990	0.6299	1.4227	0.2531	0.7725	0.6699
CAV1	0.5660	0.0144	0.8912	0.5112	0.6351	0.0334
CCL11	0.4747	0.1508	0.3332	0.2757	1.4245	0.8367
CCL2	1.6428	0.1743	**0.2161**	**0.0079**	**7.6032**	**0.0185**
CCL3	1.4448	0.4934	0.6953	0.4092	2.0781	0.3863
CCR2	0.7085	0.5419	0.8021	0.5720	0.8833	0.8901
CEBPB	1.4727	0.0269	0.5894	0.1547	2.4988	0.0238
COL1A2	1.4808	0.0005	1.9265	0.0023	0.7686	0.0250
COL3A1	1.7498	0.0160	1.8150	0.0056	0.9641	0.7933
CTGF	1.0502	0.6893	1.3088	0.0480	0.8024	0.0813
CXCR4	2.6848	0.2667	2.5817	0.3556	1.0399	0.8841
DCN	1.3845	0.1447	0.6306	0.0127	2.1953	0.0196
EDN1	0.8315	0.0207	1.0616	0.3456	0.7833	0.0137
EGF	1.6083	0.0991	2.4336	0.0250	0.6609	0.1004
ENG	1.0461	0.7206	0.6580	0.0692	1.5899	0.1135
FASLG	0.9232	0.8077	2.2583	0.3459	0.4088	0.3389
GREM1	1.1101	0.1362	0.7078	0.0145	1.5684	0.0098
HGF	0.4372	0.0009	**0.1219**	**0.0000**	**3.5852**	**0.0125**
IFNG	0.9232	0.8077	1.4227	0.2531	0.6489	0.2473
IL10	0.6114	0.9924	0.4446	0.1992	1.3751	0.4786
IL13	1.6605	0.4195	1.5333	0.0908	1.0830	0.5322
IL13RA2	0.5018	0.0323	0.6284	0.1111	0.7985	0.2892
IL1A	**9.1426**	**0.0250**	**22.2091**	**0.0307**	0.4117	0.1334
IL1B	0.8149	0.6342	0.9906	0.8526	0.8226	0.6590
IL4	1.6118	0.7286	3.0142	0.3439	0.5347	0.4425

(*Continued*)

	**Exosomes vs Untreated**		**TGFbeta vs untreated**		**Exosomes vs TGF-beta**	
IL5	0.7856	0.3181	**0.1545**	**0.0124**	**5.0844**	**0.0331**
ILK	0.7063	0.0178	0.8300	0.3770	0.8510	0.4203
INHBE	**10.9874**	**0.0161**	**26.8968**	**0.0220**	0.4085	0.0848
ITGA1	8.7288	0.7019	0.8786	0.7089	9.9350	0.3637
ITGA2	**3.9709**	**0.0024**	2.5603	0.0469	1.5509	0.1547
ITGA3	0.5088	0.0172	0.7562	0.3628	0.6728	0.2717
ITGAV	0.9610	0.7879	0.8673	0.6429	1.1080	0.7745
ITGB1	1.4364	0.0299	1.2772	0.0998	1.1246	0.3636
ITGB3	0.6096	0.0200	0.9124	0.4853	0.6682	0.0716
ITGB5	1.0796	0.5151	0.9136	0.6991	1.1818	0.4560
ITGB6	**6.5460**	**0.0017**	7.4509	0.2202	0.8786	0.5316
ITGB8	**0.1929**	**0.0145**	**0.1179**	**0.0102**	1.6359	0.3754
JUN	1.0620	0.6512	1.0433	0.7437	1.0179	0.9078
LOX	0.9665	0.8121	1.1998	0.1474	0.8056	0.1798
LTBP1	1.1981	0.1146	1.1053	0.4087	1.0839	0.4323
MMP1	**3.3610**	**0.0161**	0.7416	0.5907	**4.5322**	**0.0147**
MMP13	**14.3312**	**0.0013**	1.8575	0.0864	**7.7155**	**0.0018**
MMP14	1.3842	0.1216	0.7739	0.6900	1.7885	0.1908
MMP2	1.6081	0.0662	1.0982	0.5897	1.4643	0.1878
MMP3	42.4110	0.0096	0.5927	0.0992	71.5576	0.0093
MMP8	0.7126	0.2574	1.4758	0.1268	0.4829	0.0273
MMP9	3.2359	0.3071	0.8412	0.5845	3.8469	0.2139
MYC	1.8240	0.0150	2.3413	0.0015	0.7791	0.0976
NFKB1	0.9104	0.5020	0.7172	0.0188	1.2694	0.1947
PDGFA	0.6840	0.0546	1.1063	0.5935	0.6183	0.1044
PDGFB	0.9232	0.8077	1.4227	0.2531	0.6489	0.2473
PLAT	0.9863	0.9482	0.7119	0.1616	1.3854	0.1979
PLAU	1.2081	0.3322	1.2233	0.2320	0.9876	0.9912
PLG	0.7913	0.3020	0.4363	0.0443	1.8134	0.0568
SERPINA1	**12.3713**	**0.0147**	0.9513	0.8363	**13.0050**	**0.0155**
SERPINE1	1.0189	0.8402	1.7801	0.0849	0.5724	0.0936
SERPINH1	1.4507	0.0088	2.6688	0.0013	0.5436	0.0039
SMAD2	0.7627	0.0502	0.8805	0.2932	0.8662	0.3487
SMAD3	0.3752	0.0483	**0.1604**	**0.0098**	2.3392	0.1149

(*Continued*)

	Exosomes vs Untreated		TGFbeta vs untreated		Exosomes vs TGF-beta	
SMAD4	0.8963	0.5901	0.7615	0.0187	1.1771	0.3504
SMAD6	0.6809	0.2149	**0.3109**	**0.0045**	2.1899	0.0892
SMAD7	1.3734	0.1058	0.7495	0.3231	1.8326	0.0555
SNAI1	2.1055	0.0627	0.9437	0.8715	2.2312	0.1779
SP1	0.9925	0.8698	0.6750	0.3475	1.4703	0.2878
STAT1	1.5344	0.0957	0.7639	0.2424	2.0086	0.0086
STAT6	0.6487	0.6062	1.4970	0.8734	0.4334	0.5621
TGFB1	1.5107	0.0941	1.6242	0.1965	0.9301	0.6896
TGFB2	1.2627	0.0088	1.9774	0.0003	0.6386	0.0011
TGFB3	1.0061	0.9888	0.3261	0.0265	**3.0848**	**0.0180**
TGFBR1	1.0905	0.6675	0.5638	0.0919	1.9341	0.0711
TGFBR2	0.6924	0.1494	0.4326	0.0233	1.6007	0.0762
TGIF1	1.5901	0.0037	1.4112	0.0296	1.1268	0.2588
THBS1	0.7754	0.0959	0.8742	0.5876	0.8870	0.5287
THBS2	0.9094	0.6834	0.8009	0.6788	1.1354	0.8971
TIMP1	2.2392	0.0012	2.7349	0.0000	0.8188	0.0391
TIMP2	0.5663	0.2508	0.5551	0.2430	1.0201	0.9224
TIMP3	0.6663	0.1198	1.0161	0.9549	0.6557	0.0841
TIMP4	0.8829	0.5808	1.2689	0.0813	0.6958	0.0986
TNF	0.5817	0.3729	2.5033	0.3031	0.2324	0.1517
VEGFA	1.8100	0.0716	2.0893	0.0411	0.8663	0.6159

Bold text- highlight mRNA considered differentially expressed according to the criteria of ± 3x fold change and a *p*-value of <0.05
